# Characteristics of positive and negative autobiographical memories central to identity: emotionality, vividness, rehearsal, rumination, and reflection

**DOI:** 10.3389/fpsyg.2023.1225068

**Published:** 2023-09-13

**Authors:** Justina Pociunaite, Daniel Zimprich

**Affiliations:** Department of Developmental Psychology, Ulm University, Ulm, Germany

**Keywords:** autobiographical memory, centrality, individual differences, emotionality, vividness, rehearsal, rumination, reflection

## Abstract

**Introduction:**

Some events are remembered as more central to a person’s identity than others. However, it is not entirely clear what characterizes these autobiographical memories central to one’s identity. In this study, we examined the effects of various characteristics on centrality to identity of positive and negative memories. Characteristics such as emotionality, vividness, and how frequently a memory is retrieved and shared with others as well as ruminative and reflective self-foci were studied.

**Methods:**

The sample included 356 participants (18–92 years of age). First, participants responded to demographic questions and individual difference questionnaires. Next, they recalled memories in response to 12 emotional cue words. The cue words were balanced for emotional valence (i.e., six positive and six negative) and presented in a random order. After retrieving all memories, participants rated them regarding centrality, using the short seven-item Centrality of Event Scale and other memory characteristics, on a seven-point Likert scale. Multivariate multilevel regression was used for data analyzes, to consider multiple characteristics at the same time and account for data dependency within individual.

**Results:**

The results showed that emotionality, vividness, and frequency of memory retrieval contributed to higher centrality of memories, and employing a reflective self-focus resulted in rating memories as more central. In specific cases, these characteristics were associated differently with centrality of positive and negative memories.

**Discussion:**

Central memories can be perceived as markers in a person’s life story. The findings of this study suggest that these marker events are also highly available in a person’s memory system, by being actively emotional, visually rich, and frequently retrieved. Moreover, not only memory characteristics but also individual’s features are important to fully understand the autobiographical memory centrality.

## 1. Introduction

Autobiographical memories refer to memories of particular events that an individual has experienced in their life. Some events from a person’s life are deemed central to identity or central in short (Berntsen and Rubin, 2007). For an event to be central, it could be a turning point in an individual’s life story, it could be used as a reference point for other events, it could be considered as part of their self-identity, or it could be a combination of these concepts. Prior research has shown that autobiographical memories of events central to identity compared to their less central counterparts might show higher valence, intensity, vividness, or rehearsal (Gehrt et al., 2018). Nevertheless, this requires further replication and corroboration because prior studies focused mainly on the centrality of negative or traumatic events or compared how positive memories are different from negative ones concerning their centrality ratings (e.g., Berntsen et al., 2011). But few studies explored whether the characteristics associated with centrality of positive memories are the same as those associated with centrality of negative memories (*cf.* Boals, 2010; Pociunaite et al., 2022). Notably, data dependency within an individual warrants further attention: When a participant identifies more than one central memory, as a result, data are dependent in the sense that events and accompanying memory characteristics reported by the same person are more similar (Rubin, 2021; Pociunaite et al., 2022). Finally, while prior research found that individuals who experienced stronger emotional distress (e.g., Posttraumatic Stress Disorder (PTSD), Depression) also rated negative events as more central (Berntsen and Rubin, 2007), research into personality features not subject to emotional distress is rare.

In the present study, we aimed to identify what factors are simultaneously associated with memory centrality. Using a multivariate approach, we investigated how valence, intensity, vividness, private rehearsal, and social rehearsal are related to centrality of positive and negative memories. Analyzing these characteristics separately would bear the risk that their respective explanatory power regarding the outcome variable (i.e., centrality) is inflated (Zimprich and Wolf, 2018). The current study also adds to existing literature by employing multilevel models to take the data dependency within individuals into account by introducing random effects (e.g., Zimprich and Wolf, 2016). People may also employ ruminative and reflective self-foci to appraise their past (Trapnell and Campbell, 1999). Consequently, we examined how ruminative and reflective self-foci personality features are related to centrality of positive and negative memories. The current study extends prior research by including several characteristics studied multivariately using mixed methods to understand better the differences and similarities between positive and negative memories’ centrality.

### 1.1. Variables associated with centrality to identity

Event centrality is a tripartite concept that covers different aspects of a meaning attributed to an event and the position an event receives in a person’s identity and life story. First, an event can be central because it functions as a reference point used to compare to other events in the past or the future. Second, an event can be central in that it represents a turning point in an individual’s life, which may help explain the choices, acts, and values that followed this event. Third, an event can be central because it became a component of a person’s identity, suggesting that central memories include information relevant to identity (Berntsen and Rubin, 2006). Even though, conceptually, centrality consists of different facets, in empirical research centrality is typically measured using one combined score (e.g., Berntsen et al., 2011; Galán et al., 2017).

Prior research compared centrality of positive and negative memories in non-clinical samples and found that positive memories are rated as more central than negative ones (Berntsen et al., 2011; Rasmussen and Berntsen, 2013; Zaragoza Scherman et al., 2015, 2020; Rubin et al., 2019; Pociunaite et al., 2022). In their re-analysis of previous research on the centrality of events, Gehrt et al. (2018) found that centrality of negative events among other characteristics bivariately correlated with memory vividness (0.40), emotional intensity (0.38), and centrality of positive events (0.22). Therefore, emerging empirical findings described what can characterize central memories.

Nevertheless, it is less known whether memory characteristics are associated with centrality of positive and negative memories differently or rather similarly. Boals (2010) divided the centrality into the centrality of positive and negative memories and found that higher centrality ratings of positive memories related to negative emotions, emotional intensity, and intrusiveness, whereas centrality ratings of negative memories related to positive and negative emotions, emotional intensity, intrusiveness, and coherence and reliving, which the authors referred to as vividness. Note that in addition to examining the positive and negative memories separately, Boals (2010) asked participants to rate positive and negative emotions associated with the memory. Consequently, examining the positive and negative memories together would conceal the above-mentioned differences. In a similar study, examining a few memory characteristics at the same time with respect to centrality, Pociunaite et al. (2022) found that emotional intensity had a more substantial effect on the centrality of positive memories than on negative ones for middle-aged and older adults. Considering that centrality of positive and negative memories can be substantially different, this argues in favor of studying the two valence categories of autobiographical memories separately. Pociunaite et al. (2022) also accounted for the data dependency within individuals by studying the two-level variance. The two-level variance implied that people vary not only regarding the centrality per memory (Level-1) but also per individual (Level-2; Pociunaite et al., 2022). Therefore, centrality can be conceptualized as a memory and individual characteristic. Rubin et al. (2019) referred to this as having a self-narrative focus.

Furthermore, individual characteristics relate to how a person appraises the centrality of autobiographical memories as the relationship between autobiographical memory and identity is reciprocal (Conway, 2005). Prior studies on the centrality and individual differences predominantly examined the relation between negative events and emotional distress (e.g., depression, anxiety, PTSD), less adaptive individual traits and thinking styles (e.g., neuroticism; see Gehrt et al., 2018 for review), and posttraumatic growth (e.g., Staugaard et al., 2015; Allbaugh et al., 2016). The studies found that higher centrality of stressful/negative memories is associated with stronger feelings of distress. Fewer studies examined the centrality of positive and negative memories and more positively perceived individual traits. For instance, the associations between centrality and emotional distress, well-being, personality measures (Berntsen et al., 2011; Zaragoza Scherman et al., 2015), and self-concept clarity (Pociunaite et al., 2022) were different for positive and negative memories. Higher life satisfaction was associated with rating negative memories as less central to identity and positive ones as more central (Zaragoza Scherman et al., 2015). Moreover, well-being (feelings of happiness and support) was associated with higher centrality of positive memories and lower centrality of negative ones (Berntsen et al., 2011). A clearer self-concept (i.e., a stable personality trait that defines how clearly, consistently, confidently a person perceives themselves) related to lower centrality of negative memories, whereas centrality of positive memories was not significantly associated with self-concept clarity (Pociunaite et al., 2022). This emerging evidence suggests associations between the centrality of differently valenced memories and more positively perceived individual traits. Prior research investigated what characterizes memories that are central to identity but the research remains limited concerning the memory characteristics and centrality of positive versus negative memories and individual differences other than emotional distress measures. In the following, we focus in more detail on the variables that are associated with memory centrality.

#### 1.1.1. Emotional valence

The distinction between positive and negative valence is not the only way to conceptualize valence. Boals (2010) examined two separate measures of memory valence (positive and negative emotions) with respect to centrality and justified the need to study them separately. Ford et al. (2018) found that emotional importance was associated with both positive and negative aspects of an event, though they tested only negative events. Russell (1980) suggested that valence can be split into two independent scales of positive and negative affect. Bipolarity, which is contrary to Russell’s (1980) view, considers the positive and the negative to be the complete opposite of each other and is usually perceived as a dyad, whereas independence considers positive and negative as unipolar objects that do not necessarily contradict one another (Feldman Barrett and Russell, 1998). An event can be positive and negative at the same time, it is possible that the same event can be both highly positive and slightly negative or slightly positive and highly negative, or in a variety of different strength combinations. We do not assume that the independence and bipolarity explanations are mutually exclusive (Feldman Barrett and Russell, 1998) but in this study we focus not only on the bipolar distinction between positive and negative valence but their conceptual independence as well.

#### 1.1.2. Emotional intensity

Apart from valence, emotional intensity may also affect memory centrality. Briefly, whereas valence characterizes the quality (positive vs. negative) of an affective state, intensity refers to its magnitude, irrespective of whether it is positive or negative (Mei et al., 2018). Previous studies have shown that higher intensity is associated with a higher centrality of both positive and negative memories (Boals, 2010; Pociunaite et al., 2022). These results, however, call for further replications where participants report more than one memory (i.e., multilevel data structure), and the intensity is examined in combination with other variables (i.e., multivariate approach). The Intensity Principle suggests that—regardless of valence—individuals process intense information more efficiently than neutral information (Matlin and Stang, 1978). As a consequence, intense information has a higher probability to enter long-term memory and become central. Similarly, according to Fading Affect Bias (FAB), negative memories lose their intensity over time more quickly than positive ones, thus helping to maintain a positive self-view (Ritchie et al., 2016). With respect to centrality ratings, it is possible that positive memories are more central to identity because they do not lose their intensity as quickly as negative memories (Rubin et al., 2019; Zaragoza Scherman et al., 2020).

#### 1.1.3. Vividness

Vividness, sometimes defined as memory richness,^1^ is an important characteristic in autobiographical memory especially in conjunction with emotionality (Holland and Kensinger, 2010; Ford et al., 2012). According to Conway and Pleydell-Pearce (2000), vividness reflects the integration of the self-view and one’s goals. A memory is highly vivid when the self-view and one’s goals are either strongly integrated or strongly disjunct—vividness representing how important the event is to the identity. Regardless of the outcome (integrated or disjunct), the effort to integrate the self-view and the goals would then result in a vivid memory. With respect to centrality, Holland and Kensinger (2010) and Brown and Kulik (1977) emphasized that the vividness of autobiographical memories is related to personal involvement: The more self-relevant the memory is, the more vivid it is as well—albeit this appears to be more accurate for positive memories. This stronger association between vividness and self-relevance may also be due to the need to maintain a positive self-image (D’Argembeau and Van Der Linden, 2008; Kensinger and Ford, 2020). As such, it remains unclear for what type of memory, positive or negative, the association between centrality and vividness is more significant. We could assume that vividness of both positive and negative memories explains centrality though for potentially different reasons.

#### 1.1.4. Rehearsal

Rehearsal captures the frequency of retrieving the memory of an event, either sharing it with others or remembering it privately (Rasmussen and Berntsen, 2013; Alea et al., 2021). Even though it is possible that memories are more central to identity because they are rehearsed more often (Zaragoza Scherman et al., 2015), it is yet to be examined whether this is due to private or social rehearsal or both. The two types of rehearsal (social and private) might have different underlying processes and specific effects on memories (Skowronski et al., 2004; Ritchie et al., 2006).

Socially sharing central memories might reduce their importance (Pasupathi, 2003; Skowronski et al., 2004; Skowronski, 2011). This, however, could work differently for positive and negative events (Pasupathi, 2003; Skowronski et al., 2004). It appears as if social communication may help diminish the negative emotional intensity of negative memories, but may serve to maintain the emotional intensity of positive ones. Contrary to social rehearsal, thinking about specific events privately and repetitively may lead to rumination (Nolen-Hoeksema et al., 2008), which might result in maintaining the impact of negative memories. Gehrt et al. (2018) suggested that unconstructive repetitive thoughts could even increase the centrality of negative events. Similarly, the repetition of positive event memories could enhance their effect on centrality in line with self-enhancement theory (Skowronski, 2011). Therefore, social and private rehearsals could have potentially different effects for positive and negative memories. While either sharing or thinking about the memory could maintain the impact of positive memories, for negative memories, sharing could reduce the impact, whereas thinking repetitively about negative memories could maintain or even increase their impact.

#### 1.1.5. Rumination and reflection

We examined two personality traits: Ruminative and Reflective self-foci (Teasdale and Green, 2004). The two types of self-foci could be relevant to everyone and not only those experiencing emotional disturbances. Rumination is usually considered a way of thinking in a repetitive manner leading to maladaptive consequences. It is usually rather abstract without considering a specific situation. On the other hand, reflection could be the opposite of rumination and has an end-point of reasoning, such as solving a problem (Trapnell and Campbell, 1999; Habermas et al., 2021). Trapnell and Campbell (1999) defined it as: “Rumination provides a summary conception of self-attentiveness motivated by perceived threats, losses, or injustices to the self. Reflection provides a summary conception of self-attentiveness motivated by curiosity or epistemic interest in the self” (p. 297).

Some studies already proposed the importance of ruminative self-focus to autobiographical memory (Teasdale and Green, 2004; Finnbogadóttir and Thomsen, 2013; Rubin et al., 2014, 2019; Gehrt et al., 2018). Allbaugh et al. (2016) found that the centrality of a single stressful event positively related to adaptive and maladaptive types of rumination. Berntsen et al. (2011) found that neuroticism, conceptually similar to rumination in its concern with negative emotions and proneness to ruminate, was positively associated with the centrality of positive and negative memories. However, Rubin et al. (2011) found no significant correlation between neuroticism and centrality. These findings could be replicated employing a different study design (see below). The positive individual characteristics (e.g., reflective self-focus) still warrant further research concerning the centrality of positive and negative memories. We cannot necessarily assume that reflection is the direct opposite of rumination because a person can have a combination of both styles.

### 1.2. The present study

Prior research indicated the potential contribution of various autobiographical memory characteristics concerning centrality of positive and negative memories. To the best of our knowledge, the abovementioned memory and individual characteristics have yet to be studied in a multivariate approach that would explain their relative contribution to centrality. Moreover, few studies investigated that each memory characteristic, including centrality, can also vary for each individual differently. The two-level variance implies that an individual’s remembering style, such as ratings of memories as emotional, vivid, or rehearsed more often, would also be related to the ratings of memory centrality (Zimprich and Wolf, 2016).

Centrality to identity reflects a memory characteristic but it could also reflect an individual trait of an inclination to recall events in a self-narrative focus. In the present study, participants reported more than one memory of central events, thus, data were statistically dependent as events reported by the same person are potentially more similar. Analyzing memory centrality using multilevel models has the additional advantage that the characteristics can be examined on the level of event memories (Level 1) and on the level of individuals (Level 2). The present research aimed to identify (1) whether the memory characteristics of emotionality, vividness, and rehearsal (private and social) predict the centrality of positive and negative memories. We expect some memory characteristics to show different effects on centrality for different types of memories (i.e., emotionality, vividness, and rehearsal), whereas others such as intensity are assumed to have similar tendencies for positive and negative memories. As another goal, we examined (2) whether the individual self-foci of rumination and reflection contribute to the centrality of autobiographical memories. Based on prior research, we expect a more substantial relationship of rumination with the centrality of negative memories than positive ones (Berntsen et al., 2011). However, we also expect differences concerning the centrality of positive and negative memories. The reflection does not constitute the polar opposite of rumination, and the relationship to the centrality of positive and negative memories can be more mixed.

Notably, this study comprised a more varied sample concerning participant’s age, important for the centrality research (Zaragoza Scherman et al., 2020; Pociunaite et al., 2022). Studies that comprised only younger adults found fewer differences between the centrality of positive and negative memories (e.g., Rasmussen and Berntsen, 2009). Additionally, the majority of studies examining the centrality of the event asked participants to retrieve one negative/traumatic/stressful and one positive event. Therefore, our study complements the prior research also by investigating multiple event memories per person, using a different retrieval method (12 emotion-related words), and accounting for two-level conceptualizations (e.g., centrality as a memory characteristic and centrality as an individual characteristic). Pociunaite et al. (2022) used a similar design to study centrality to identity and the influence of memory and individual characteristics, this study also aims to replicate some of their results.

## 2. Methods

### 2.1. Participants

The sample comprised 356 participants. In order to participate in the study, participants were required to have a working knowledge of German and be 18 years of age or older. Participants were recruited using promotional flyers and posters, contacting third-age university groups, and word of mouth methods. The sample included 64.6% (*n* = 230) women; 34.3% (*n* = 122) men.^2^ Participants’ age ranged from 18 to 92 years, with an average age of 43.1 years (SD = 21.4 years). The majority of the sample had at least 12 years of education (i.e., German Abitur): 41.6% (*n* = 148); or higher: 37.6% (*n* = 134). 44.9% (*n* = 160) and 43.5% (*n* = 155) of the participants reported being either in a relationship or single, respectively. A large number of participants were students (33.7%, *n* = 120); were employed (28.93%, *n* = 103); or in retirement (27.3%, *n* = 97). Participants rated their subjective health from *very good* (1) to *inadequate* (5), the sample average score was 2.05 (SD = 0.83). The sample mood mean score amounted to 3.94 (SD = 0.82) indicating good mood on average as tested using the Self-Assessment Manikin (SAM; Bradley and Lang, 1994) prior to memory retrieval.

### 2.2. Materials

#### 2.2.1. Centrality of event

The centrality of event scale (CES; Berntsen and Rubin, 2006) is a self-report scale that evaluates how central a certain memory is to identity and life story, whether it becomes a reference or a turning point. We used a short version of this questionnaire that consists of seven items (e.g., “This event permanently changed my life”). Each item was evaluated on a five-point Likert scale from *totally disagree* (1) to *totally agree* (5). Originally, the authors reported internal consistency to be ranging between 0.87 and 0.92. Cronbach’s alpha in the current sample amounted to 0.93 on the memory level and 0.96 on the individual level, again showing good internal consistency.

#### 2.2.2. Memory cues

We chose the cues for memory elicitation based on the Circumplex (Russell, 1980) and Vector (Talarico et al., 2004) models of emotions. In a first step, we extracted words that are positive or negative and well balanced on arousal, with priorities given to words that overlap between Circumplex and Vector models. Second, we translated these words into German—however, in some cases, a direct translation to the German language was not possible or if it was, their emotional weight (the valence and arousal) was different from the English ones. Finally, we used the Berlin Affective Word List (BAWL-R; Võ et al., 2009) to guide us through the process of balancing the cues based on their valence and arousal scores (Sheldon et al., 2020). The BAWL-R is a list of 2,200 German words that provides the ratings of a word’s valence and arousal on a five-point Likert scale. The final cue choice was motivated by their similarity in valence and arousal to the English words. We selected 12 emotion-related adjectives and nouns as cue words: six positive and six negative emotions, of which three words represented low-arousal and three words represented high-arousal emotions. Positive low-arousal emotion words were satisfied (*zufrieden*), calm (*ruhig*), glad (*froh*), and positive high-arousal emotion words were happy (*glücklich*), astonished (*erstaunt*), delighted (*erfreut*). Negative low-arousal emotion words were listless (*lustlos*), sad (*traurig*), lonely (*einsam*), and negative high-arousal emotion words were distressed (*Sorge*), afraid (*Angst*), angry (*Ärger*).

#### 2.2.3. Memory characteristics

Participants assessed several memory characteristics for each reported memory. We asked for participant’s *age* at the time of event. Subtracting this value from the current participant’s age allowed us to define a variable of how recent a memory was. Other memory characteristics (i.e., positive/negative valence, intensity, vividness, private and social rehearsal) were rated on seven-point Likert scales: *How* positive *would you rate the event today? Not at all* (1) *– Very positive* (7)*; How* negative *would you rate the event today? Not at all* (1) *– Very negative* (7)*; How emotionally* intense *would you rate the memory today? Not at all* (1) *– Very intense* (7)*; How* vividly *do you remember this event? Not at all* (1) *– As clear as it just happened* (7)*; How often do you* talk *about this event? Almost never* (1) *– Very often* (7)*; How often do you* think *about this event? Almost never* (1) *– Very often* (7). All questions were presented in a random order, except for the positive and negative valence evaluation which were rated jointly in order to avoid confusion.

#### 2.2.4. Rumination and reflection

The rumination-reflection questionnaire (RRQ; Trapnell and Campbell, 1999) is a self-report questionnaire used to evaluate dispositional self-focus, or put otherwise, the overall emotion regulation strategies a person is using, irrespective of a specific event. The questionnaire consists of two 12-item scales: Rumination and Reflection. The Rumination scale had items that read for example “I spend a great deal of time thinking back over my embarrassing or disappointing moments,” whereas the Reflection scale consisted of the items similar to “My attitudes and feelings about things fascinate me.” Each item was rated on a five-point Likert scale ranging from *Strongly disagree* (1) and *Strongly agree* (2). Higher overall scores indicated stronger rumination and reflection. The original study reported Cronbach’s alpha of 0.90 and 0.91 for rumination and reflection scales, respectively (Trapnell and Campbell, 1999). In the current study, the rumination scale Cronbach’s alpha amounted to 0.89, whereas Cronbach’s alpha of reflection scale amounted to 0.85.

### 2.3. Procedure

The study was carried out online using the www.unipark.de platform. At the time of data collection, approval by the ethical board of Ulm University (Ulm, Germany) was not required for studies in which data collection was completely anonymous. Data was collected online. Participants were provided with detailed information about the study participation and data protection.

They could only participate after giving their consent (opt-in approach). Data was collected completely anonymous and in accordance with the Declaration of Helsinki. After providing informed consent, participants reported their demographic characteristics (see *Participants*) and filled out the individual differences’ questionnaire (see *Materials*). The memory retrieval process followed. In the instruction, participants were presented with a autobiographical memory description and were asked to retrieve memories older than 1 year to avoid a recency effect (Conway, 2005).^3^ Afterwards, participants were shown the 12 cue words in a randomized order one after the other. Based on each cue word, participants were asked to come up with a memory. To avoid lingering, participants had 3 min in total to evoke a memory and take a brief note for themselves to refresh their recollection later. Directly following this procedure, people were re-confronted with the memories they retrieved to the cue words and asked to rate them according to *CES* and *memory characteristics* (see above). At the end of the study, participants were able to share feedback and participate in a book voucher lottery worth 10 EUR, psychology undergraduates also had an option to receive course credit. The data collection process spanned from December 2020 until July 2021.

### 2.4. Analytical approach

To analyze the data, we applied multilevel multiple regression. Multilevel analysis is well-suited to account for the memories’ nesting within individuals (i.e., each person retrieved up to 12 memories). Multiple regression was necessary to consider more than one independent variable at the same time. CES^4^ was treated as the dependent variable. The analyzes were carried out in a stepwise manner, starting with a fixed effects model, followed by the addition of random effects, which would imply the variation of CES not only between memories but between individuals as well. In subsequent models the memory and individual characteristics were added.

To differentiate between positive and negative memories based on the cues, we used two indicator variables:


$$\begin{array}{l} I_{p}\left(y_{ij}\right)=\{\begin{array}{cc} 1 & \mathrm{if}\;\mathrm{the}\;\mathrm{valence}\;\mathrm{of}\;y_{ij}\;\mathrm{is}\;\mathrm{positive}\;\\ 0 & \mathrm{otherwise} \end{array}\;\mathrm{and}\\ I_{n}\left(y_{ij}\right)=\{\begin{array}{cc} 1 & \mathrm{if}\;\mathrm{the}\;\mathrm{valence}\;\mathrm{of}\;y_{ij}\;\mathrm{is}\;\mathrm{negative}\;\\ 0 & \mathrm{otherwise} \end{array}\text{.} \end{array}$$

A mixed-effects model for CES ratings that differentiates between positive and negative autobiographical memories can then be written as

$$y_{ij}=I_{p}\left(y_{ij}\right)\left(\beta_{0}+u_{i}+x_{i}^{\prime }\beta +e_{ij}\right)+I_{n}\left(y_{ij}\right)\left(\gamma_{0}+w_{i}+x_{i}^{\prime }\gamma +e_{ij}\right)$$

where *β*_0_ is the fixed CES intercept for positive memories, *u_i_* is the random deviation of individual from the fixed intercept, **x***_i_* is a vector of covariates, **β** is a vector of regression coefficients linking the covariates and the CES (for positive memories), and *e_ij_* is a residual. Analogously, *γ*_0_ is the fixed CES intercept for negative memories, *w_i_* is the random deviation of individual from the fixed intercept, **x***_i_* is a vector of covariates, **γ** is a vector of regression coefficients linking the covariates and the CES (for negative memories). The detailed analytic approach using cue valence (positive and negative) as indicators is described in Pociunaite et al. (2022).

Memory characteristics were group-mean centered for the Level-1 (memory level) analyzes, whereas Level-2 (individual level) characteristics were grand-mean centered. We also calculated context means for the memory characteristics to be able to use them on the individual level. For example, to test if a person’s higher scores on intensity or vividness overall would influence the individual’s tendency of centrality. All data analyzes were carried out using SAS Proc Mixed procedure (SAS Institute Inc, 2014). We provide −2 × the log-likelihood (−2LL) and Akaike’s Information Criterion (AIC) to indicate model fit. See also Vrieze (2012) for a description of AIC properties. Additionally, we calculated R^2^ values on Level 1 and Level 2 for models one to three using the approach proposed by Rights and Sterba (2021).

## 3. Results

### 3.1. Preliminary analyzes

Participants reported a total of 3,979 memories. The average number of memories recalled was 11.18, ranging from 5 to 12. Mean CES score for this sample was 2.81 (SD = 1.19), with a range from 1 to 5. Table 1 provides a detailed description of other memory characteristics. Notably, the differences in total frequency between private and social rehearsal were significant (*p* < 0.001), Cohen’s *d* = 0.39. The mean sample rumination score amounted to 3.32 (SD = 0.77), the mean score of reflection was 3.55 (SD = 0.68). We also estimated the correlations between memory characteristics on the individual (Table 2) and memory (Table 3) levels. The tables show a number of large (> 0.5) and medium-sized correlations (> 0.3). A better participant’s mood shortly before memory reporting was significantly associated with lower centrality (*r* = −0.107, *p* = 0.044) and lower negativity of negative memories, and higher positivity and lower negativity of positive memories.

**Table 1:** Descriptive values of the memory variables.

	Positive	Negative	Total
	M (SD)	M (SD)	M (SD)
1 Centrality	2.90 (1.22)	2.73 (1.17)	2.81 (1.19)
2 Memory age (months)	32.25 (18.12)	30.37 (18.45)	31.31 (18.31)
3 Positivity	5.79 (1.56)	2.35 (1.63)	4.08 (2.34)
4 Negativity	1.61 (1.33)	4.78 (1.81)	3.19 (2.24)
5 Intensity	4.75 (1.74)	4.41 (1.81)	4.59 (1.78)
6 Vividness	5.19 (1.57)	4.96 (1.65)	5.08 (1.61)
7 Private rehearsal	3.87 (1.85)	3.53 (1.83)	3.70 (1.85)
8 Social rehearsal	3.20 (1.85)	2.78 (1.69)	2.99 (1.78)

**Table 2:** Correlation matrix of variables on the individual’s level.

	1	2	3	4	5	6	7	8	9	10	11	12
1 Centrality	-	0.176**	0.225**	−0.107*	0.114*	0.076	0.354**	0.112*	0.559**	0.431**	0.648**	0.475**
2 Rumination	−0.044	-	0.324**	−0.292**	−0.365**	−0.293**	−0.065	0.192**	0.138*	0.049	0.114*	−0.019
3 Reflection	0.126*	0.324**	-	−0.042	−0.166*	−0.168*	0.042	0.035	0.156*	0.182**	0.058	0.049
4 SAM	0.017	−0.292**	−0.042	-	0.073	0.031	0.072	−0.103*	−0.069	0.038	−0.078	0.042
5 Age	0.340**	−0.365**	−0.166**	0.073	-	0.826**	0.009	−0.065	0.111*	0.212**	0.215**	0.238**
6 Memory age	0.240**	−0.354**	−0.201**	0.111*	0.861**	-	−0.034	0.015	0.142*	0.207**	0.265**	0.287**
7 Positivity	0.275**	−0.049	0.115*	0.104*	−0.037	−0.018	-	−0.490**	0.134*	0.010	0.240**	0.331**
8 Negativity	0.125*	0.066	−0.123*	−0.123*	0.063	0.057	−0.466**	-	0.359**	0.313**	0.177**	−0.029
9 Intensity	0.601**	−0.101	0.009	0.049	0.373**	0.325**	0.452**	0.053	-	0.691**	0.632**	0.414**
10 Vividness	0.513**	−0.094	0.041*	0.147*	0.293**	0.262**	0.434**	−0.007	0.778**	-	0.521**	0.370**
11 Private rehearsal	0.621**	−0.127*	−0.049	0.062	0.348**	0.363**	0.257**	0.155*	0.685**	0.621**	-	0.661**
12 Social rehearsal	0.546**	−0.166*	−0.053	0.132*	0.291**	0.305**	0.209**	0.155*	0.540**	0.468**	0.783**	-

*p < 0.05; **p < 0.001. Contextual means are used for memory characteristics. Lower diagonal is for memories elicited with positive cues, Upper diagonal for memories elicited with negative cues.

**Table 3:** Correlation matrix of variables on the memory level.

	1	2	3	4	5	6	7	8
1 Centrality	-	−0.023	0.081**	0.135**	0.422**	0.349**	0.429**	0.280**
2 Memory age (months)	−0.034	-	0.035	−0.001	0.118**	0.093**	0.214**	0.188**
3 Positivity	0.141**	0.005	-	−0.613**	−0.065*	−0.034	−0.028	0.036
4 Negativity	0.009	0.033	−0.655**	-	0.342**	0.264**	0.228**	0.096**
5 Intensity	0.405**	0.057*	0.312**	−0.037	-	0.587**	0.579**	0.389**
6 Vividness	0.295**	0.101**	0.298**	−0.029	0.561**	-	0.482*	0.341**
7 Private rehearsal	0.404**	0.169**	0.247**	−0.023	0.590**	0.489**	-	0.548**
8 Social rehearsal	0.344**	0.140**	0.215**	−0.056*	0.443**	0.377**	0.591**	-

*p < 0.05; **p < 0.001. Lower diagonal is for memories elicited with positive cues, Upper diagonal for memories elicited with negative cues.

Based on cue valence, the memories were divided into two groups: positive and negative. Out of the total number of 3,979 memories, 1998 memories were reported in response to positive cues, whereas 1981 memories were reported in response to negative ones, participants reported 5.74 and 5.73 positive and negative memories on average. The cue manipulation checks according to valence and arousal suggested that valenced cues served as a powerful technique for retrieving differently valenced types of memories.^5^ For the positive cues, positivity rating mean was 5.79 (SD = 1.56), whereas the mean of negativity was 1.61 (SD = 1.33). These values were significantly and negatively correlated *r* = −0.66, *p* < 0.001. Similarly, for the negative cues, negativity averaged to 4.78 (SD = 1.81) across memories, meanwhile positivity mean was 2.35 (SD = 1.63), again significantly and negatively correlated *r* = −0.61, *p* < 0.001. This shows that positive and negative cues prompted memories rated higher in positivity and negativity, respectively. In the following models, we present the results based on positive and negative cues indicating CES of positive and CES of negative memories.

### 3.2. Multilevel modeling results

#### 3.2.1. Model 0

In this model, we estimated CES fixed effects. The CES fixed intercept amounted to 2.81 (*p* < 0.001), which denotes the mean of each person’s individual mean of CES ratings. The model fit -2LL was 12,711 and the AIC amounted to 12,713. After including random intercept effects, model fit improved considerably: -2LL = 11,902, AIC = 11,906. Random intercept variance for the CES was 0.44 (SE = 0.04, *p* < 0.001), residual variance: 0.99 (SE = 0.02, *p* < 0.001). The random intercept variance captures the variation of individual intercepts around the fixed intercept, which, because it is significant, implies that individuals show reliably different average CES ratings. The CES intraclass correlation for positive memories amounted to 0.35, whereas for negative to 0.32, implying that 35 and 32% of the CES variance is between individuals for positive and negative memories, respectively. Moreover, other memory characteristics also showed substantial intraclass correlations: Intensity 0.227, vividness 0.270, social rehearsal 0.281, private rehearsal 0.257, positivity 0.026, negativity 0.031.

#### 3.2.2. Model 1

**Figure 1:**
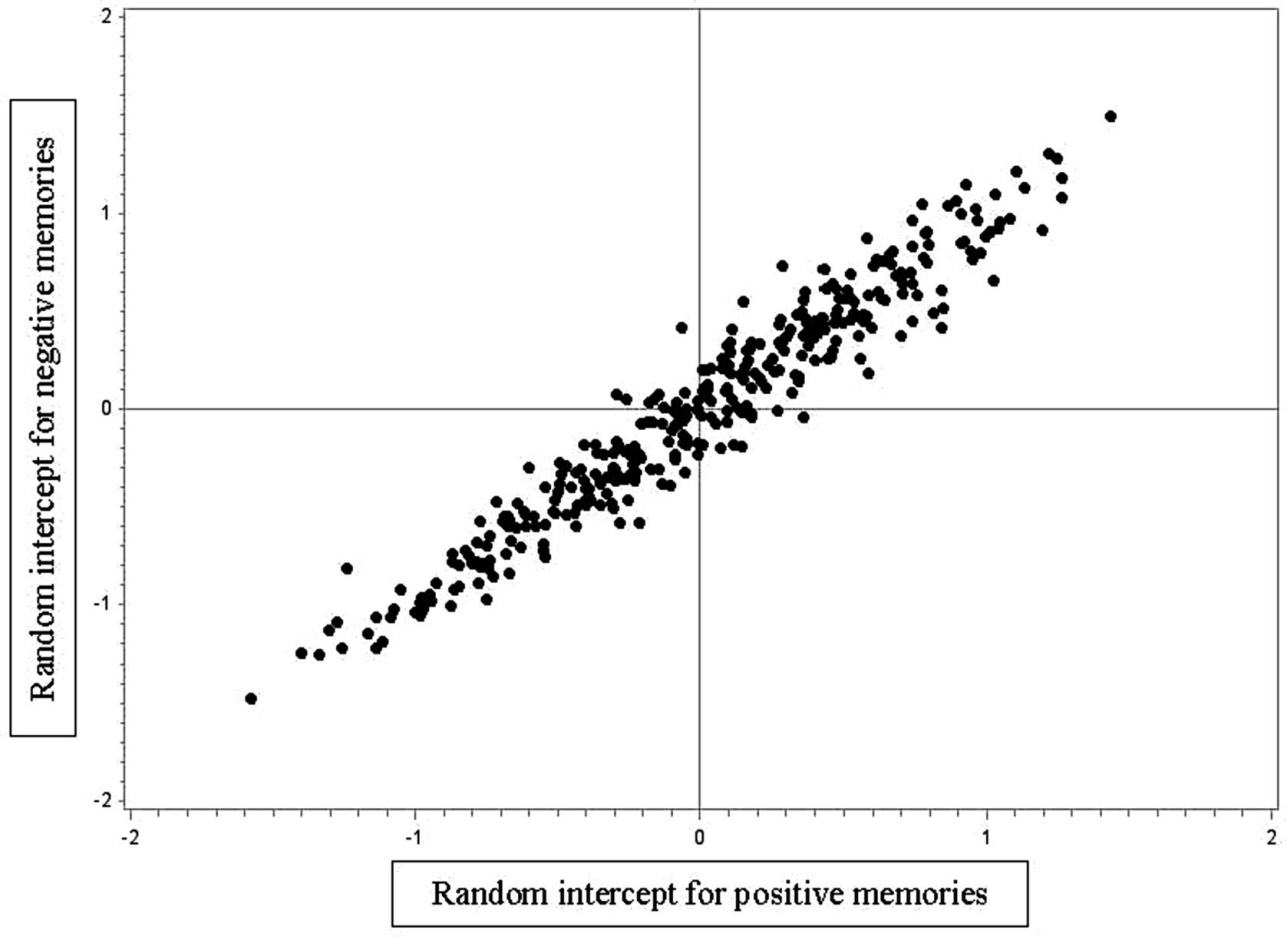
The correlation (*r* = 0.89) between random intercepts of centrality for positive (x-axis) and negative (y-axis) memories.

In addition to Model 0, we estimated fixed and random effects at the baseline model for positive and negative memories by including the indicator variables and control variables of participant’s age and gender. Higher participant age was associated with higher centrality for both positive and negative memories, though for positive memories this association was larger (*p* < 0.001). The fixed CES intercept for positive memories was 2.89 (*p* < 0.001), whereas for negative memories it amounted to 2.73 (*p* < 0.001). The difference between the two estimates was significant (*p* < 0.001). Moreover, the two random intercepts correlated significantly (*r* = 0.89, *p* < 0.001), see Figure 1 for the graphic representation of the correlation. Note that this correlation does not depict observed individual centrality means of positive and negative autobiographical memories, but is based on the random intercepts (i.e., latent variables) that capture the individuals’ deviation from the fixed intercept. The correlation implies a strong and positive association between individual differences in CES ratings of positive and negative autobiographical memories. Estimating CES for positive and negative cues memories improved the model fit (change of -2LL = 194, change of AIC = 188).

#### 3.2.3. Model 2

In this next model, we included memory characteristics (Level 1) as predictors of CES. The control variables used in the model were participant’s age, gender, and memory age (i.e., recency). The significance of memory age (i.e., recency) indicated that the older the memory, the lower the centrality ratings. Higher participant’s age predicted higher centrality ratings. Table 4 shows parameter estimates for each model in detail. In essence, memory characteristics showed largely similar tendencies: The higher the memory ratings on valence, intensity, vividness, and private and social rehearsals, the higher the centrality of the memories as well. Social rehearsal and negativity were stronger associated with centrality of positive memories than negative ones, while vividness was stronger associated with centrality of negative memories than positive ones. Other memory characteristics showed similar effects for the centrality of two types of memories (positive and negative). Based on parameter estimates values, intensity (B_pos_ = 0.147; B_neg_ = 0.169) and private rehearsal (B_pos_ = 0.173; B_neg_ = 0.197) were the strongest predictors of centrality. The correlation analyzes of observed estimates could potentially support this relationship, showing that intensity and rehearsal correlated the strongest with centrality, compared to other memory characteristics as presented in Table 3. The correlation between centrality and intensity was 0.405 (*p* < 0.001), private rehearsal 0.404 (*p* < 0.001) for positive memories, and 0.422 (*p* < 0.001) and 0.429 (*p* < 0.001) for negative memories. Random variation remained significant across the two types of cues, and the model fit improved considerably from 11,708 to 9,078 for -2LL, and 11,718 to 9,088 for AIC. The explained variance R^2^ increased compared to Model 1, also indicating a better model fit.

**Table 4:** Multilevel modeling results, where centrality is the dependent variable.

	Model 1		Model 2		Model 3	
	Positive	Negative	Positive	Negative	Positive	Negative
Fixed effects						
Intercept	2.891*	2.725*	2.793*	2.912*	2.801*	2.930*
Age^a^	0.013*	0.004*	0.011*	0.006*	0.014*	0.008*
Gender^a^	−0.021	0.029	−0.026	−0.014	−0.015	−0.013
Positivity^b^			0.085*	0.085*	0.088*	0.086*
Positivity^a^					0.262*	0.164*
Negativity^b^			0.079*	0.025	0.080*	0.022
Negativity^a^					0.083	0.099
Intensity^b^			0.147*	0.169*	0.148*	0.168*
Intensity^a^					0.161*	0.128*
Vividness^b^			0.026	0.077*	0.029	0.078*
Vividness^a^					−0.025	−0.018
Private rehearsal^b^			0.173*	0.197*	0.167*	0.195*
Private rehearsal^a^					0.208*	0.321*
Social rehearsal^b^			0.098*	0.051*	0.097*	0.054*
Social rehearsal^a^					0.060	0.022
Memory Age^b^			−0.017*	˗0.015*	−0.018*	−0.015*
Memory Age^a^					−0.012*	−0.011*
Rumination^a^					0.082	0.082
Reflection^a^					0.163*	0.146*
Random Effects						
Pos Intercept variance	0.44*		0.44*		0.20*	
Neg Intercept variance	0.44*		0.46*		0.20*	
Pos and Neg Corr	0.89*		0.98*		0.96*	
Pos Residual variance	0.94*		0.56*		0.56*	
Neg Residual variance	0.97*		0.66*		0.66*	
Model fit						
-2LL	11,708		9,078		8,912	
AIC	11,718		9,088		8,922	
R^2^ Level 1	0%	0%	33%	40%	33%	40%
R^2^ Level 2	15%	3%	12%	5%	54%	54%

*p < 0.05; ^a^refers to variables being centered between persons (Level 2); ^b^refers to variables being centered within persons (Level 1); Estimates in gray signify significant differences between the parameter estimates for positive and negative memories; We also examined the influence of participant’s mood concerning memory centrality. The variables were not associated significantly: B_pos_ = ˗0.022 and B_neg_ = ˗0.036 (*p* > 0.10). The model including participant’s mood ratings as an independent variable (grand-mean centered) is presented in Supplementary material A.

#### 3.2.4. Model 3

In the following model, we included memory characteristics on the individual level (Level 2) using contextual means,^6^ contextual means were created by averaging memory level units within a level-2 unit. Here we accounted for the fact that memory characteristics can serve as an individual’s characteristics as well. On the memory level (Level 1), the tendencies remained the same as in Model 2: higher memory characteristics ratings were associated with higher CES; the differences between positive and negative memories remained consistent with Model 2.

**Figure 2:**
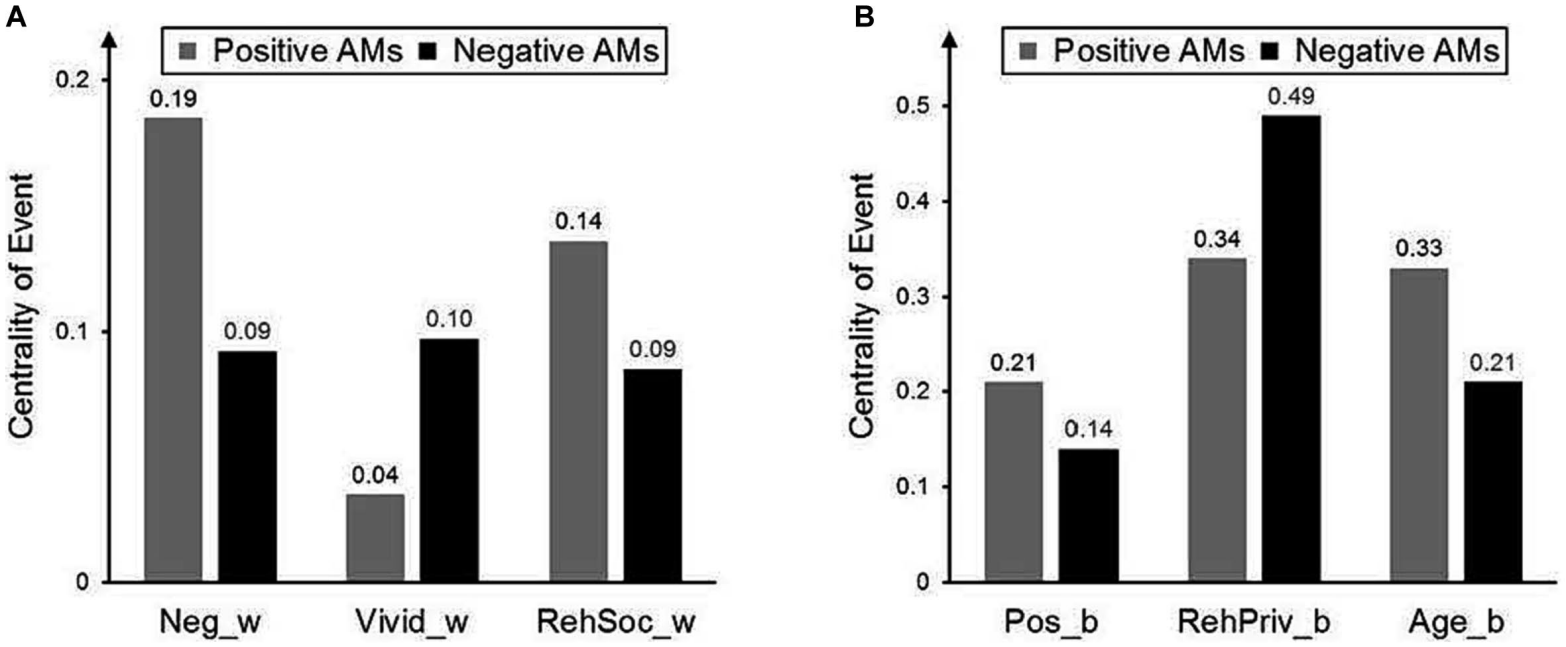
Standardized beta weights for those variables, where parameter estimates differ significantly between the positive and negative autobiographical memories. Panel A: Memory level (Level 1) effects. Panel B: Individual level (Level 2) effects. Variables have been standardized based on Wang et al. (2019). Panel A: Neg_w = Negativity (centered within person–Level 1), vivid_w = Vividness (centered within person–Level 1), RehSoc_w = Social rehearsal (centered within person–Level 1); Panel B: Pos_b = Positivity (centered between person–Level 2), RehPriv_b = Private rehearsal (centered between person–Level 2); Age_b = Participant’s age (centered between person–Level 2).

Positivity, intensity, and private rehearsal were memory characteristics on the individual level that significantly predicted centrality of event for both positive and negative memories. The results can be read as follows: If a person on average retrieved higher intensity scores by one-unit as compared to the sample mean, it associated with the higher scores of centrality by 0.161 for positive, and by 0.128 for negative memories. If a person rated intensity higher by one unit above the person’s average, the centrality was rated as higher by 0.148 for positive memories, and by 0.168 for negative ones. Vividness, negativity, and social rehearsal were insignificant on the individual level (Level 2), implying that it influences centrality regarding memories but individual differences in the said characteristics did not have an effect on centrality.^7^ Regarding the differences between positive and negative memories on the individual level, private rehearsal had a stronger impact on centrality of negative memories than on positive ones, while positivity had a stronger impact on centrality of positive memories than negative ones. Overall, positivity, intensity and private rehearsal consistently related to centrality: For positive and negative memories, and for both memory and individual level measures. To denote the significant differences between positive and negative memories, in Figure 2 we presented the parameter estimates of variables that showed significant differences between positive and negative memories while using standardized coefficient values. The standardization was done based on Wang et al. (2019), using a person-mean-SD standardization method instead of the global standardization. The differences are even more pronounced when using standardized values.

In Model 3, we also included individual characteristics such as rumination and reflection. Higher ratings of reflection were significantly associated with individuals rating centrality as higher for both positive and negative memories. Rumination was not a significant predictor of centrality for positive nor for negative memories. Random effects remained significant and the model fit improved from 9,078 to 8,912 (−2LL), and from 9,088 to 8,922 (AIC), the variance explained by this model increased substantially as can be seen from the increased *R*^2^ values.

## 4. Discussion

In the current study, we examined which autobiographical memory and individual characteristics are associated with the centrality of autobiographical memories. We focused on memory characteristics such as emotionality, vividness, and frequent memory sharing and private remembering. Our findings demonstrate that the characteristics are, in fact, associated with centrality, and some influence positive and negative memories differently. Additionally, we found that how people rate centrality depends not only on memory characteristics, but also on the individual. More precisely, centrality is linked to what kind of memories people retrieve (e.g., higher in positivity, intensity, and frequently rehearsed) and a specific type of self-focus (i.e., reflection). The current study findings suggest that central memories are also highly available: emotionally active, visually rich, and frequently brought up in people’s minds or conversations. In the following, we first discuss how memory characteristics and centrality are related and link them to prior literature. Then, we focus on individual differences in both reporting memory characteristics and individual features concerning centrality.

### 4.1. Memory characteristics influencing centrality

Considering valence, our findings suggested that if a memory is rated as emotional, either positively or negatively, it is also rated as central to identity. The design manipulation substantiated that positivity and negativity had correspondingly higher rates for the positive and negative cue memories. In addition to positive and negative categorization, by introducing two additional variables we also accounted for the possible independence of positivity and negativity (Russell, 1980). We found that both characteristics are important when rating centrality of memories. Our findings also confirmed that the centrality of positive memories was higher than the centrality of negative ones, which is in line with previous literature (e.g., Berntsen et al., 2011). Independent positivity and negativity scales provided additional information, suggesting that central events can have positive and negative features (Ford et al., 2018).

Furthermore, central memories are also actively re-experienced memories (i.e., emotionally intense). Regardless of the valence type, intensity significantly and consistently associated with memory centrality (Talarico et al., 2004; Boals, 2010; Pociunaite et al., 2022). Prior studies on centrality found that positive and negative central memories are also intense (Boals, 2010; Pociunaite et al., 2022) and that intensity is one of the strongest predictors of centrality (of negative memories; Gehrt et al., 2018). Research on autobiographical memory emotionality (Matlin and Stang, 1978; Comblain et al., 2005) also emphasized that intensity is one of the essential aspects of important autobiographical memories compared to their valence. Importance can be perceived as conceptually similar to memory centrality. Together with the current study results, this implies that for a memory to be salient in a person’s life story, it should be emotionally active. Our findings also support the idea that valence and intensity represent different aspects of memory emotionality: Intensity covers the magnitude of the affective state, whereas valence covers the quality of the affective state (Mei et al., 2018). In summary, intensity could be equally important, or even more important, for the centrality of positive than negative memories (Boals, 2010; Pociunaite et al., 2022). Our study complements prior research by examining how the centrality of positive and negative memories is linked to intensity, as prior research focused mainly on negative events and their relationship between centrality and intensity (Gehrt et al., 2018).

In the current study, compared to intensity, the association between vividness and centrality was less substantial, contrary to the conclusions in the review by Gehrt et al. (2018), though this review mostly bivariately focused on negative events and their centrality. The relationship between vividness and centrality can be attenuated due to other, stronger predictors, which in our case was intensity. Our findings supported this by demonstrating that vividness significantly related with centrality when considered in isolation, but its relationship to centrality changed when other characteristics were added. Moreover, vividness was more strongly associated with the centrality of negative memories compared to positive ones. Looking closer at the construct of centrality, especially central events being reference points to other events, we observe some similarities to a directive memory use. The directive memory use refers to memories guiding the person’s behaviors (Pillemer, 2003). Similarly, prior research found that negative memories used for directive functions are also negative in valence (Rasmussen and Berntsen, 2009; Wolf and Zimprich, 2015; Wolf and Demiray, 2019) and highly vivid (Pillemer, 2003; Rasmussen and Berntsen, 2009). Keeping the negative memories vivid and central can help a person use them to guide future behaviors (Rasmussen and Berntsen, 2009; Wolf and Zimprich, 2015; Wolf and Demiray, 2019). These similarities between centrality and functional memory use are worth investigating in future research. Our findings also propose that vividness acts as a memory characteristic only, whereas other characteristics can transfer into a general individual’s way of remembering. We come back to the topic of individual differences below.

Based on our findings, we observed that frequently sharing and especially frequently thinking of memories are linked to higher centrality, even when controlling for other memory characteristics such as emotionality and vividness. First, private rehearsal was used more frequently than social rehearsal, which supports the findings of Alea et al. (2021). Second, both types of rehearsal were significantly associated with centrality. However, thinking of memories in private was more strongly associated with centrality than sharing the memories with others. This finding suggests that there could be different mechanisms associated with private and social rehearsals (Ritchie et al., 2006). Third, socially rehearsed positive memories had a stronger association with centrality than negative ones, implying the benefit of social rehearsal to maintain the impact of the memory for a positive event (Pasupathi, 2003; Skowronski et al., 2004). Finally, Zaragoza Scherman et al. (2015) suggested that rehearsal is one of the most prominent explanations of centrality. To the best of our knowledge, the distinction between private and social rehearsals has not been investigated before with a focus on centrality.

Furthermore, in the current study, we observed that more recent memories are more central, which follows some previous literature (e.g., Rubin et al., 2019; Pociunaite et al., 2022), but other studies had the opposite findings (e.g., Berntsen and Bohn, 2010). A potential explanation lies in that the temporal distance effects might be less linear and perhaps better explained by other phenomena, such as a reminiscence bump (Wolf and Zimprich, 2020). We also controlled for participant’s age, which showed that with increased participant’s age, the centrality increased as well. We know from previous studies that age groups showed different tendencies regarding centrality (Pociunaite et al., 2022). The positivity effect describes the appreciation of emotional information that changes with age (Carstensen et al., 1999). According to positivity effect, with increasing age, people might favor positive information over negative information (Hamilton and Allard, 2021). Therefore, the significant age associations with ratings of centrality while controlling for valence may be considered in light of potential differences in emotional information processing by older adults. Concerning insignificant gender effects on centrality ratings in this study, Boals (2010) and Sotgiu (2019) suggested that the differences between genders might stem from the types of events reported, not necessarily individual differences. However, this study was not equipped to answer this question and requires future research concerning gender comparisons.

In summary, our findings demonstrate that memories central to identity are more emotional, vivid, and frequently talked about and thought about. While positivity, intensity, and private rehearsal related to centrality to identity of positive memories similarly, others, such as negativity, vividness, and social rehearsal, were differently associated with the centrality of positive and negative memories. This strengthens our motivation to focus not only on negative but also on positive event memories that are central to identity, as different mechanisms could motivate the processing of positive and negative memory information (e.g., Rasmussen and Berntsen, 2009). Our findings also stress that central memories can be explained by both memory characteristics and an individual’s remembering style, to which we turn next.

### 4.2. Individual characteristics influencing centrality

Our findings align with previous research that suggested that memory characteristics could be seen as individual characteristics (Luchetti et al., 2016; Rubin et al., 2019; Rubin, 2021). Individuals’ average ratings of positivity, intensity, and private rehearsal, showed significant associations with centrality. Thus, if a person tends to rate memories as more positive and intense and think of them more often, compared to other participants, they would also rate their memories as more central to identity. The significant random intercept variation suggested that people, in general, could view events from their past as being more (or less) central to their identity. Similarly, most memory characteristics demonstrated a significant variation between individuals as judged by their intraclass correlations. This, in addition to random intercept variation, indicates that people have their remembering style across several events. In addition, our study confirmed the need to account for the two-level structure within the nested data, as indicated by the explained variance on Level 1 and Level 2 and its increase. We examined which individual characteristics could be associated with higher memory centrality.

Rating positive memories as more central to identity could be associated with the theory of maintaining a positive self-view (Skowronski et al., 2014; Janssen et al., 2022) or self-enhancement (Janssen et al., 2022). People generally tend to see themselves as positive and use self-enhancement functions for this purpose (Janssen et al., 2015). Moreover, some individuals maintain the emotional information intact more than others (Rubin et al., 2012; Kensinger and Ford, 2020). The finding that positivity and intensity serve as individual characteristics influencing centrality may support the idea that reporting more emotional memories represents an individual’s relationship with emotionality, suggesting a more pronounced experience of emotions by some (Rubin et al., 2012). Therefore, if a person experienced life through a more emotional lens, this would also relate to rating memories as more central in general. In addition, we found that a tendency to rehearse memories frequently is associated with higher centrality. People who think about their memories more often also tend to rate them as more central, on average. This could support the reciprocity idea between memory and personality (Conway, 2005). If individuals frequently retrieve their memories, it may help to integrate them into a life story and keep them central, essentially developing a self-narrative focus.

Lastly, our findings showed that a self-focus of reflection but not rumination (sometimes referred to as emotion regulation strategies) relate to a person’s tendency to rate memories as more central to identity, which partially aligns with prior research (Allbaugh et al., 2016; Del Palacio-Gonzalez and Berntsen, 2018, 2020). This suggests that having a more constructive rather than obstructive self-focus may influence centrality more. To put it differently, reflecting on one’s past constructively, not in a repetitive manner (i.e., ruminative), could be associated with better integration of central events into a person’s life-story and better appreciation of the meaning each event might bare. Importantly, this is not limited to positive events, and includes both types of central memories: positive and negative.

Rumination did not significantly relate to centrality ratings, which is contrary to the assumptions made by Gehrt et al. (2018). Finnbogadóttir and Thomsen (2013) proposed several explanations for the inconsistent findings regarding rumination, namely, different measures for Ruminative Thinking, differences in samples, or focusing on different rumination phases. Another possible explanation of these findings may lie in the associations between rumination, centrality, and the strength of emotional responses. Based on our findings, centrality is associated with a more pronounced emotional response, whereas rumination, while unpleasant at the time, might reduce a person’s emotional response in the long term. These differences in the emotional response strength might potentially explain an insignificant association between rumination and centrality.

### 4.3. Limitations and future directions

In the current study, we complemented prior autobiographical memory research regarding the characteristics influencing centrality to identity. However, a few limitations of our study could be addressed in future studies. First, the use of emotional cues could affect what kind of memory is retrieved (specific or overgeneral) more than other types of cues; while specificity is related to other characteristics such as valence or intensity (Hallford et al., 2021), there is no clear indication that specificity is related to centrality to identity. Nonetheless, this study could not control for the type of event. Moreover, our results underscore that it is essential to consider the memory cueing method and its impact on result generalization because different cueing methods (e.g., free recall) could show more pronounced differences between different valence memories and centrality to identity (e.g., Boals, 2010; Pociunaite et al., 2022). Second, the sample of this study showed relatively low levels of rumination, which could have contributed to insignificant findings (Habermas et al., 2021). Notwithstanding, the large sample size of this study covering the entire adult lifespan could adequately represent the general population and potentially its levels of rumination. Third, due to correlational nature of the data this study was not equipped to infer the causality of the memory and individual characteristics with respect to centrality. Finally, the list of possible memory characteristics is not exhaustive, therefore, the ratings with respect to additional characteristics (e.g., physical reaction, life-script correspondence) could extend our study findings, however, including a long list of characteristics could also increase participant’s burden. Similarly, the specific content coding of the event was not within the scope of the current study, but could extend our study findings in future examinations.

Future studies could extend our research by studying how memory characteristics interplay while impacting centrality. For instance, previous literature has provided some empirical evidence for the bivariate or trivariate interaction analyzes between the memory characteristics [e.g., valence-intensity (Talarico et al., 2004), vividness-intensity (Kensinger and Ford, 2020), valence-vividness-intensity (Ford et al., 2012)]. However, one would need to recognize that the complexity of the design would require extra considerations. Moreover, disentangling each predictor’s unique and shared contributions to centrality (i.e., commonality analyzes) could also extend the findings of our study. In addition, future studies using multilevel structural equation models could explore specific pathways, for instance, whether rumination predicts rehearsal or emotionality, which, in turn, predict centrality (Boals, 2010); memory centrality may predict rehearsal, which in turn would predict emotionality (Rubin et al., 2011). Finally, memories are usually dynamic; thus, their reconstruction can change over time (Conway, 2005). Prior research on positive and negative affect changes (Ritchie et al., 2016) has already tapped into this topic. However, a more detailed description of how memory characteristics change over time and how their influence on centrality might also change would be necessary to address.

## 5. Conclusion

The present study identified some memory and individual characteristics that are associated with autobiographical memory centrality. This study extended prior research by investigating positive and negative memories and personality features extending beyond emotional distress measures. Our findings confirmed that autobiographical memories central to identity are emotional, vivid, and frequently rehearsed, but in some cases, it depends on the memory valence. Additionally, memory characteristics can be operationalized as an individual’s remembering styles that, together with reflective self-focus, impact centrality to identity. For instance, individuals remembering their past more emotionally and appraising it more adaptively also recall their past events as more central to identity. The causal relationships between the characteristics at hand and centrality to identity are yet to be defined. However, at the moment, we can conclude that the autobiographical memories central to identity are frequently rehearsed and emotionally and vividly re-experienced.

## Data availability statement

The datasets presented in this article are not readily available due to institutional data protection considerations. Requests to access the datasets and the syntax used for data analysis should be directed to justina.pociunaite@uni-ulm.de.

## Ethics statement

Ethical approval was not required for the studies involving humans as data were collected voluntarily and anonymously regarding everyday phenomena. The study was conducted in accordance with the local legislation and institutional requirements and the Declaration of Helsinki. The participants provided their informed consent to participate in this study.

## Author contributions

JP wrote the original draft of the manuscript and was responsible for data curation, formal analysis, methodology, and project administration. DZ reviewed and edited the manuscript and supervised the project. JP and DZ were involved in the conceptualization and data visualization. All authors contributed to the article and approved the submitted version.

## Conflict of interest

The authors declare that the research was conducted in the absence of any commercial or financial relationships that could be construed as a potential conflict of interest.

## Publisher’s note

All claims expressed in this article are solely those of the authors and do not necessarily represent those of their affiliated organizations, or those of the publisher, the editors and the reviewers. Any product that may be evaluated in this article, or claim that may be made by its manufacturer, is not guaranteed or endorsed by the publisher.

## Footnotes

^1^Vividness has been conceptualized differently in the literature, e.g., as visual imagery (Greenberg and Knowlton, 2014), visual clarity (Sutin and Robins, 2007), number of perceptual details (Janssen et al., 2022), or amount of reliving (Barzykowski and Mazzoni, 2022).

^2^Four (1.12%) participants defined their gender as other, due to the small number of the participants in the latter group the *other* group in gender variable was treated as missing.

^3^It usually takes time for recent memories to be integrated into the long-term memory system. Recent memories are subject to inhibition or different processing from the important memories that are already stored in the long-term memory system [see Conway (2005), for a review].

^4^We conducted additional analyses to test centrality as a latent variable defined by seven questionnaire items. The results using latent variable were largely similar to centrality computed as an average score. We opted for the latter option to have more flexibility in the results analyses program and presentation.

^5^We compared the significant differences between memory ratings on valence and intensity elicited by different cue categories. The nested within the individual nature of this data was accounted for. The positivity fixed intercept for positive cues was 5.79, for negative it was 2.36, and the estimated difference between the two intercepts was significant. The negativity fixed intercept for positive cues was 1.61, whereas for the negative cues it was 4.77, and the difference was also significant. Finally, the intensity fixed intercept for high arousal cues was 4.70, whereas for low arousal cues it was 4.47, the difference between the two estimates was significant. This shows that cues that varied in valence and arousal prompted the memories that are also varied in valence and intensity, accordingly. Moreover, we checked for possible nesting within cues. Random effects were small, especially compared to the individual’s random effects. Hence, we did not include it in further analyses but rather accounted for the cues in a different way (i.e., the indicator).

^6^Taking into consideration the Lüdtke bias (Lüdtke et al., 2008), we computed a number of analyses using latent means instead of the contextual, however, it yielded largely similar results. Hence, we used contextual means for centering, which is conceptually and statistically simpler (Bryk and Raudenbush, 1992; Zimprich and Wolf, 2018).

^7^To investigate this relationship in more detail, we conducted additional analysis, where vividness was the sole predictor of centrality. In this case, vividness significantly predicted centrality on the memory (Level 1) and individual (Level 2) levels (*p* < 0.001). It confirms the importance of multivariate analysis as a linear regression can lead to incomplete results.
