# Supplementary material for: Characteristics of positive and negative autobiographical memories central to identity: emotionality, vividness, rehearsal, rumination, and reflection
Source: Front Psychol. 2023 Sep 13;14:1225068. doi: 10.3389/fpsyg.2023.1225068 (PMC10534006; doi:10.3389/fpsyg.2023.1225068)
Supplement: Supplementary file 1 [file Table_1.DOCX]

**Supplementary Material A**

*Multilevel modelling results, where centrality is the dependent variable;*

*Model 4 included participant’s mood that was rated shortly prior to memory retrieval*

|  |  | **Model 4** | |
| --- | --- | --- | --- |
|  |  | Positive | Negative |
| *Fixed Effects* | |  |  |
| Intercept |  | 2.801***** | 2.929***** |
| Age^a^ |  | 0.015***** | 0.008***** |
| Gender^a^ |  | -0.019 | -0.019 |
| Positivity^b^ |  | 0.088***** | 0.086***** |
| Positivity^a^ |  | 0.264***** | 0.168***** |
| Negativity^b^ |  | 0.080***** | 0.023 |
| Negativity^a^ |  | 0.081 | 0.096 |
| Intensity^b^ |  | 0.148***** | 0.168***** |
| Intensity^a^ |  | 0.158***** | 0.123***** |
| Vividness^b^ |  | 0.029 | 0.078***** |
| Vividness^a^ |  | ˗0.020 | ˗0.001 |
| Private rehearsal^b^ | | 0.167***** | 0.195***** |
| Private rehearsal^a^ | | 0.205***** | 0.316***** |
| Social rehearsal^b^ | | 0.097***** | 0.054***** |
| Social rehearsal^a^ | | 0.063 | 0.026 |
| Memory Age^b^ | | ˗0.018***** | ˗0.015***** |
| Memory Age^a^ | | ˗0.012***** | ˗0.011***** |
| Rumination^b^ |  | 0.075 | 0.071 |
| Reflection^a^ |  | 0.163***** | 0.146***** |
| Mood^a^ |  | ˗0.022 | ˗0.036 |
|  |  |  |  |
| *Random Effects* | |  |  |
| Pos Intercept variance | | 0.20***** | |
| Neg Intercept variance | | 0.20***** | |
| Pos & Neg Corr | | 0.96***** | |
| Pos Residual variance | | 0.56***** | |
| Neg Residual variance | | 0.66***** | |
|  |  |  |  |
| *Model fit* |  |  |  |
| -2LL |  | 8921 | |
| AIC |  | 8931 | |

*Note.* **p* < 0.05. ^a^ refers to variables being centered between persons (Level 2); ^b^ refers to variables being centered within persons (Level 1); Estimates in grey signify significant differences between the parameter estimates for positive and negative memories.

**Results**

Participant’s mood was not significantly associated with ratings of centrality, nevertheless, the tendency points to better mood being linked to lower ratings of memory centrality.
